# The dynamics of pyrethroid resistance in *Anopheles arabiensis* from Zanzibar and an assessment of the underlying genetic basis

**DOI:** 10.1186/1756-3305-6-343

**Published:** 2013-12-06

**Authors:** Christopher M Jones, Khamis A Haji, Bakari O Khatib, Judit Bagi, Juma Mcha, Gregor J Devine, Matthew Daley, Bilali Kabula, Abdullah S Ali, Silas Majambere, Hilary Ranson

**Affiliations:** 1 Department of Vector Biology, Liverpool School of Tropical Medicine, Pembroke Place, Liverpool L3 5QA, UK; 2 Zanzibar Malaria Control Programme, Mwanakwerekwe, P.O. Box 407, Stone Town, Zanzibar, Tanzania; 3 QIMR Berghofer Institute of Medical Research, 300 Herston Road, Herston Brisbane QLD 4006, Australia; 4 National Institute for Medical Research, Amani Research Centre, P.O. Box 81, Muheza, Tanzania; 5 Ifakara Health Institute, PO Box 78373, Dar es Salaam, Tanzania

**Keywords:** *Anopheles arabiensis*, Zanzibar, Pyrethroid resistance, P450s, Gene expression

## Abstract

**Background:**

The emergence of pyrethroid resistance in the malaria vector, *Anopheles arabiensis*, threatens to undermine the considerable gains made towards eliminating malaria on Zanzibar. Previously, resistance was restricted to the island of Pemba while mosquitoes from Unguja, the larger of the two islands of Zanzibar, were susceptible. Here, we characterised the mechanism(s) responsible for resistance on Zanzibar using a combination of gene expression and target-site mutation assays.

**Methods:**

WHO resistance bioassays were conducted using 1-5d old adult *Anopheles gambiae s.l.* collected between 2011 and 2013 across the archipelago. Synergist assays with the P450 inhibitor piperonyl-butoxide were performed in 2013. Members of the *An. gambiae* complex were PCR-identified and screened for target-site mutations (*kdr* and *Ace-1*). Gene expression in pyrethroid resistant *An. arabiensis* from Pemba was analysed using whole-genome microarrays.

**Results:**

Pyrethroid resistance is now present across the entire Zanzibar archipelago. Survival to the pyrethroid lambda-cyhalothrin in bioassays conducted in 2013 was 23.5-54.3% on Unguja and 32.9-81.7% on Pemba. We present evidence that resistance is mediated, in part at least, by elevated P450 monoxygenases. Whole-genome microarray scans showed that the most enriched gene terms in resistant *An. arabiensis* from Pemba were associated with P450 activity and synergist assays with PBO completely restored susceptibility to pyrethroids in both islands. *CYP4G16* was the most consistently over-expressed gene in resistant mosquitoes compared with two susceptible strains from Unguja and Dar es Salaam. Expression of this P450 is enriched in the abdomen and it is thought to play a role in hydrocarbon synthesis. Microarray and qPCR detected several additional genes putatively involved in this pathway enriched in the Pemba pyrethroid resistant population and we hypothesise that resistance may be, in part, related to alterations in the structure of the mosquito cuticle. None of the *kdr* target-site mutations, associated with pyrethroid/DDT resistance in *An. gambiae* elsewhere in Africa, were found on the islands.

**Conclusion:**

The consequences of this resistance phenotype are discussed in relation to future vector control strategies on Zanzibar to support the ongoing malaria elimination efforts on the islands.

## Background

A major obstacle facing malaria control is pyrethroid resistance in the malaria vectors, *Anopheles gambiae* and *Anopheles funestus*[1,2]. Populations of highly resistant *An. gambiae* span West Africa [3,4] while pockets of resistance continue to emerge throughout the east of the continent [5-7]. With alternative compounds for long-lasting insecticide nets (LLINs) and indoor residual spraying (IRS) still in the development pipeline, selection for resistance looks certain to continue unabated unless effective resistance management strategies are implemented.

The search for genetic markers underpinning insecticide resistant phenotypes in natural insect populations poses significant challenges. This is exemplified in *An. gambiae* which displays a profile of resistant phenotypes across Sub-Saharan Africa. Identifying and tracking resistance-associated genetic markers is identified as a priority in the World Health Organisation’s (WHO) Global Plan for Insecticide Resistance Management (GPIRM) [1]. Resistance to pyrethroids and DDT is strongly associated with the *knockdown resistance* (*kdr*) target-site mutations (L1014F/S) in the voltage gated sodium channel [8-10] but these mutations cannot account for all the variation observed in resistance bioassays and indeed, are absent from some resistant populations [11,12]. Pyrethroid resistance is mediated in part at least by the elevated expression of metabolic gene families (e.g. P450 monooxygenases/glutathione-S-transferases/esterases) and transcriptional profiling of resistant and susceptible *An. gambiae* followed by functional validation has begun to pinpoint individual genes responsible [13,14].

Substantial reductions in malaria have been made in Zanzibar through (i) the administration of artemisin combination therapies (ACTs), (ii) island wide distribution of LLINs and (iii) IRS campaigns using the pyrethroid, lambda-cyhalothrin [15,16]. In 2010, pyrethroid resistance was reported in *Anopheles arabiensis* from the Zanzibar archipelago [17] but curiously, resistance was confined to the smaller island of Pemba, which lies 50 km north of the larger island Unguja. It was uncertain whether the discrepancy in resistance levels between Pemba and Unguja was due to different selective forces acting on the islands.

In this study, we set out to characterise the mechanism(s) responsible for the ongoing selection of resistance on Zanzibar using DNA and RNA-based approaches. Furthermore, we continued to monitor for phenotypic resistance across the islands between 2011 and 2013 in view of ongoing strategies for managing the emergence of pyrethroid resistance in Zanzibar.

## Methods

### Insect collections

**Figure 1:**
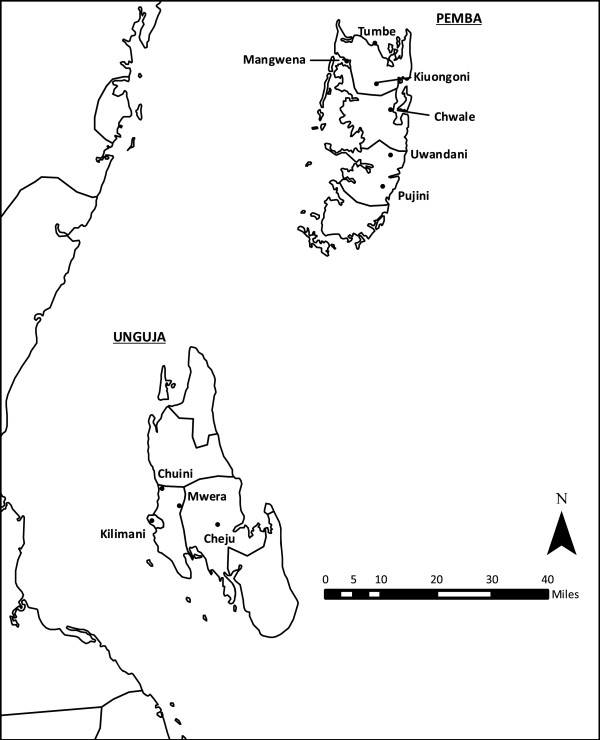
***Anopheles *** **larval collection sites from Zanzibar.**

*Anopheles* larvae were collected from breeding pools across Unguja and Pemba during the main rainy season (April/May) between 2011 and 2013 (Figure 1). The sites were chosen to represent the typical diversity of breeding grounds present on the islands and their inclusion varied year to year depending on the number of larvae available. Four sites were sampled from Unguja: Mwera, Chuini, Kilimani, and Cheju. Breeding pools from Mwera and Chuini were generally from irrigated rice fields while Cheju is situated within the Jozani forest consisting of varied tropical vegetation. Kilimani is situated on the coastal plain of the major capital Stone Town where the tide frequently submerges breeding sites resulting in brackish water. The breeding sites from Pemba were located in Pujini, Kiungoni, Uwandani, Mangwena and Tumbe. The majority of mosquitoes from these were sampled within rural inland settings with the exception of Tumbe which lies close to the north coast of the island. Mosquitoes were sampled from different pools to reduce sampling bias from families. Larvae were transported to insectaries at ZMCP and reared to adults under 27°C ± 2°C.

### Phenotypic bioassays

Resistance assays were performed on one to five day old non blood-fed female adult *An. gambiae* using WHO susceptibility tests [18]. In 2011 and 2013, *An. gambiae* were exposed to a selection of the following insecticide-impregnated papers: lambda-cyhalothrin (0.05%), permethrin (0.75%), DDT (4%) and bendiocarb (0.1%). Insufficient numbers of larvae were collected from some sites to test all insecticides and in these sites we prioritised lambda-cyhalothrin as this insecticide has been sprayed widely across Zanzibar as part of the IRS campaign since 2006 (ZMCP unpublished report, 2011). Control assays using non-treated papers were run alongside all tests. Mortality was scored 24 h post-exposure. In 2013, we performed additional synergism assays with the P450 inhibitor piperonyl butoxide (PBO). Mosquitoes were pre-exposed to PBO (4%) for one-hour and then transferred to the insecticide exposure tube for an additional hour. Mortality was scored 24 h after exposure to insecticide. Control assays exposing mosquitoes to PBO only were run in each synergist assay.

In 2012, a more comprehensive assessment of lambda-cyhalothrin resistance was performed to quantify the difference in resistance between *An. gambiae* from Pemba and Unguja. The lethal concentration for 50% mortality (LC_50_) was determined from two sites on Unguja (Chuini & Mwera) and Pemba (Mangwena & Tumbe). Mosquitoes were exposed to insecticide impregnated papers at a range of five concentrations of lambda-cyhalothrin (0.005% - 0.2%) for one hour as described above.

### An. gambiae complex identification and resistance-associated SNPs

Members of the *An. gambiae* species complex were determined using allele specific-PCR [19]. Target-site mutations in the sodium channel (resistance to pyrethroids/DDT) and acetylcholinesterase (*ace*-*1*) (resistance to carbamates/organophosphates) were screened using TaqMan allelic discrimination assays [20,21].

### Whole genome microarrays

To investigate whether differential gene expression underscored the resistant phenotype on Pemba, two whole genome microarray experiments were performed in 2011 and 2012. The emergence of pyrethroid resistance on Pemba, but apparent absence from Unguja, permitted a direct comparison of RNA extracted from mosquitoes from each island. However, in 2011 we were constrained by the number of mosquitoes collected from Unguja and presence of *An. gambiae s.s.* from our main site Kilimani. Therefore, surviving mosquitoes exposed to lambda-cyhalothrin were compared directly with the insecticide susceptible *An. arabiensis* lab-colony from Mozambique (MOZ) [11]. In 2012, the experimental design was extended to include mosquitoes from (i) Pemba (Mangwena), (iii) Unguja (Chuini) and (iii) a susceptible field strain of *An. arabiensis* from Dar es Salaam (DAR). Four day old, non blood-fed *An. arabiensis* from Ilala and Kinondoni in Dar es Salaam were stored in RNAlater® 24 h post-exposure to insecticide and display no resistance to pyrethroids or DDT. The inclusion of DAR provided an independent susceptible population to improve the strength of resistant gene expression association. Three biological replicates from each strain were integrated into an interwoven loop design as this has been shown to provide robust power in detecting differences in gene expression [22].

The *An. gambiae* 15 k whole genome microarray was used in all experiments [14]. Total RNA was extracted from batches of 8–10 female mosquitoes using the Ambion RNA4PCR kit. RNA was labelled with cy3 and cy5 dyes and hybridised to the microarray chip according to the protocol described previously [14]. RNA and cRNA quantity and quality were assessed using a Nano-drop spectrophotometer (Nano-drop Technologies) and Bioanalyser (Agilent) respectively.

### Microarray analysis

The direct comparison between PEM and MOZ was analysed using GeneSpring v.11.5 (Agilent) which applies a standard t-test to normalised raw fluorescence values. Following normalisation with LIMMA [23] the MAANOVA package in R [24] was applied to the loop design as used previously [14]. The two independent experiments from 2011 and 2012 were considered together, providing independent datasets from which improved confidence in identifying genuine candidate genes could be made. Probes were filtered based on the hypothesis of significance (q < 0.05) and under the hypothesis that expression should be consistently greater in the resistant compared to the susceptible populations (i.e. PEM > UNG/DAR). Microarray data are available in the ArrayExpress database (http://www.ebi.ac.uk/arrayexpress) under accession number E-MTAB-2075 (PEM vs. MOZ direct comparison) and E-MTAB-2074 (PEM vs. UNG/DAR loop design).

### Patterns of resistance-associated gene expression on Zanzibar

The expression levels of candidate genes for pyrethroid resistance from the microarray analysis were assessed using quantitative reverse transcription PCR (qRT-PCR) following the MIQE guidelines [25].

qPCR was performed on mosquitoes collected from 2012 and 2013 with three objectives:

I. To independently validate gene expression in *An. arabiensis* from 2012 in the microarray experiments.

II. To determine gene expression patterns of candidate genes from Pemba and Unguja in collections made in 2013.

III. To independently validate the expression of a subset of genes up-regulated in Pemba which are putatively part of the hydrocarbon synthesis pathway.

Whole female mosquitoes were collected 24 h post-exposure to insecticide or non-treated papers, briefly chilled (10–20 min) and stored in RNAlater® according to the manufacturer’s instructions. Total RNA was extracted from *An. arabiensis* using PicoPure® RNA Isolation Kit (Invitrogen) and treated with DNase I (Qiagen). Three biological replicates of RNA extracted from ten mosquitoes were used for the 2012 samples to replicate the microarray experiments. The following year, four-five biological replicates of five mosquitoes per RNA sample were used. RNA quantity and quality was checked using a Nano-Drop spectrophotometer (Nano-drop Technologies) and Bioanalyser (Agilent) respectively. cDNA was synthesised from ~0.5-1 μg of RNA using oligo(dT)_20_ (50 μM) and SuperScript III (200 U) (Invitrogen) and purified through a DNA-binding column (Qiagen).

Multiple pairs of exon-exon spanning primers for target and control genes were designed *in silico* against the *Anopheles gambiae* PEST sequence (Taxon ID 180454) using primer-BLAST (NCBI). The PCR efficiency, dynamic range and specificity of the primer pair were calculated from running a standard curve over a five-fold dilution series (input cDNA from ×5^-1^ to ×5^-5^). The primer sequences, amplicon length, efficiencies and gene location are given in Additional file 1.

PCR reactions (20 μl) were performed on the MXPro qPCR system (Agilent) with 10 μl Brilliant III SYBR Green (Agilent), 300 nM of primers, 2.5 μl of input cDNA (diluted 100-fold) and the total volume made up with sterile-distilled water. The thermal profile used throughout consisted of 95°C for 10 min followed by 40 cycles of 95°C for 10s and 60°C for 10s. Melt curves were run after each PCR to ensure the specificity of the amplified products. Three technical replicates were run for each sample and no template controls were run for each gene.

### Data analysis of gene expression

The data were pre-processed prior to analysis. A Cq value of 35 was considered our limit of detection (LOD) and any samples that failed to amplify above this threshold were given a value of 35. Outliers were defined as those values which give a standard deviation above 0.5 from the three technical replicates and were removed from the dataset.

Once pre-processed the Cq values were adjusted according to the efficiency of the primer pair. The qPCR repeats were averaged and data normalised against the average values for the ribosomal S7 protein (AGAP010592) and ubiquitin (AGAP007927). The relative quantities of each sample were calculated and log-transformed to normalise the distribution of the data for parametric statistical analysis [26]. A one-sided t-test at p < 0.05 was used to assign significance between treatments.

### Copy number analysis of P450 candidates

Several insects have adapted to selection from insecticides by evolving variations in gene copy number (otherwise known as copy number variation (CNV)) (reviewed in [27]). Quantitative PCR (qPCR) using SYBR Green was used to determine whether the elevated expression of *CYP4G16*, *CYP6Z2*, and *CYP6Z3* in *An. arabiensis* from Pemba is due to gene amplification. The quantity of genomic DNA (gDNA) extracted from individual *An. arabiensis* from Unguja and Pemba was analysed using a PicoGreen® assay [28] and diluted to 1 ng/μl. Internal exonic primers were designed for the target genes *CYP4G16*, *CYP6Z2* and *CYP6Z3* and for two internal reference genes, elongation factor (AGAP005128) and glucose-6-phosphate dehydrogenase (AGAP010739) (Additional file 1). The efficiency, dynamic range and specificity of each primer pair were assessed by performing a standard curve on a dilution series of DNA (five-fold dilutions from ×5^-1^ to ×5^-5^). SYBR Green qPCR reactions were performed on the Stratagene MXPro using the exact same conditions as RT-qPCR (see above) with the exception that 2.5 μl input gDNA was added to each reaction. To improve the power of detecting a real difference in copy number between resistant and susceptible mosquitoes, copy number analysis was performed on eight individual *An. arabiensis* which died at the lowest concentrations of insecticide exposure (0.001-0.005%% λ-cyhalothrin) from Unguja and ten individuals which survived the highest concentrations (0.1-0.2% λ-cyhalothrin) from Pemba. The average gene copy number was calculated using the ΔΔCq method described previously [29].

### Sodium channel sequencing

Exon 20 of the sodium channel harbours key residues for pyrethroid and DDT binding in the so-called ‘binding pocket’ and mutations in this region confer resistance in other insect pests [30]. To determine whether pyrethroid resistance was due to previously unidentified SNPs in this region of *An. arabiensis*, a ~400 bp fragment of Exon20 was amplified from gDNA extracted from individuals on Pemba and Unguja collected in 2012. PCR reactions were undertaken in 25 μl total volumes consisting of 0.5U Taq Polymerase (KappaBiosystems), 0.5 μl dNTPs (10 mM) and 400 nM of forward and reverse primers. Two alternative splice forms of this exon occur in *An. gambiae* (Exon c/d) and two sets of primer pairs were designed in flanking introns to amplify each variant (Additional file 1). Thermal cycling parameters were 95°C for 2 min followed by 40 cycles of 95°C for 30 s, 55°C for 30 s, and 72°C for 30 s and a final extension step of 72°C for 5 mins. Samples for sequencing were performed by Macrogen (Amsterdam) with the forward and reverse primers used as the sequencing primers.

### CYP4G16 cloning and sequencing

The full length *CYP4G16* was amplified from cDNA synthesised from pools of five *An. arabiensis* individuals from Pujini (Pemba) and Mwera (Unguja). The PCR products were amplified using the forward and reverse primers AGAP001076_F1/R1 (Additional file 1) and cloned into the pJET 1.2/blunt cloning vector (Fermentas). Two clones from each strain were sequenced (Core Genomic Facility, University of Sheffield) with the pJET forward and reverse primers as well as three nested forward primers (AGAP001076_F2/F3/F4) (Additional file 1).

## Results

### Study area and current distribution of An. gambiae complex on Zanzibar

According to the sites we visited during the study (Figure 1) *An. arabiensis* is the dominant vector on Zanzibar (Table 1). The exception was in Kilimani, situated within the main urban area of Stone Town and adjacent to the coast, where we identified 68% *An. gambiae s.s.*, 17% *An. arabiensis* and 15% *An. merus* in 2011. Two years later all mosquitoes were scored as *An. arabiensis* from this site (N = 66). In Tumbe, on the north coast of Pemba, 39% of *Anopheles* were typed as *An. merus* (N = 134) consistent with their salt-water adaptation. However, the following year, we did not identify a single *An. merus* from Tumbe (N = 71); 98% samples from this site were *An. arabiensis,* 2% *An. gambiae*.

**Table 1:** **Species identification of a subset of *An. gambiae s.l. *collected between 2011 and 2013 on Zanzibar**

**Year**	**Island**	**Site**	**No. tested**	***An. arabiensis***	***% An. arabiensis***	***An. merus***	***% An. merus***	***An. gambiae***	***% An. gambiae***	An. quad*	% An. quad*
2011	Unguja	Kilimani	78	13	16.7	12	15.4	53	67.9	0	0.0
	Pemba	Chwale	24	23	95.8	0	0.0	0	0.0	0	0.0
		Kiungoni	24	24	100.0	0	0.0	0	0.0	0	0.0
		Uwandani	24	24	100.0	0	0.0	0	0.0	0	0.0
2012	Unguja	Chuini	72	72	100.0	0	0.0	0	0.0	0	0.0
		Mwera	110	110	100.0	0	0.0	0	0.0	0	0.0
	Pemba	Tumbe	340	206	60.6	134	39.4	0	0.0	0	0.0
		Mangwena	286	284	99.3	4	1.4	0	0.0	0	0.0
2013	Unguja	Chuini	102	102	100.0	0	0.0	0	0.0	0	0.0
		Mwera	71	71	100.0	0	0.0	0	0.0	0	0.0
		Kilimani	66	66	100.0	0	0.0	0	0.0	0	0.0
		Cheju	28	16	57.1	0	0.0	6	21.4	6	21.4
	Pemba	Pujini	75	75	100.0	0	0.0	0	0.0	0	0.0
		Kiungoni	92	92	100.0	0	0.0	0	0.0	0	0.0
		Uwandani	69	69	100.0	0	0.0	0	0.0	0	0.0
		Tumbe	71	70	98.6	0	0.0	1	1.4	0	0.0

*Anopheles quadriannulatus.

### Insecticide resistance and synergism

**Figure 2:**
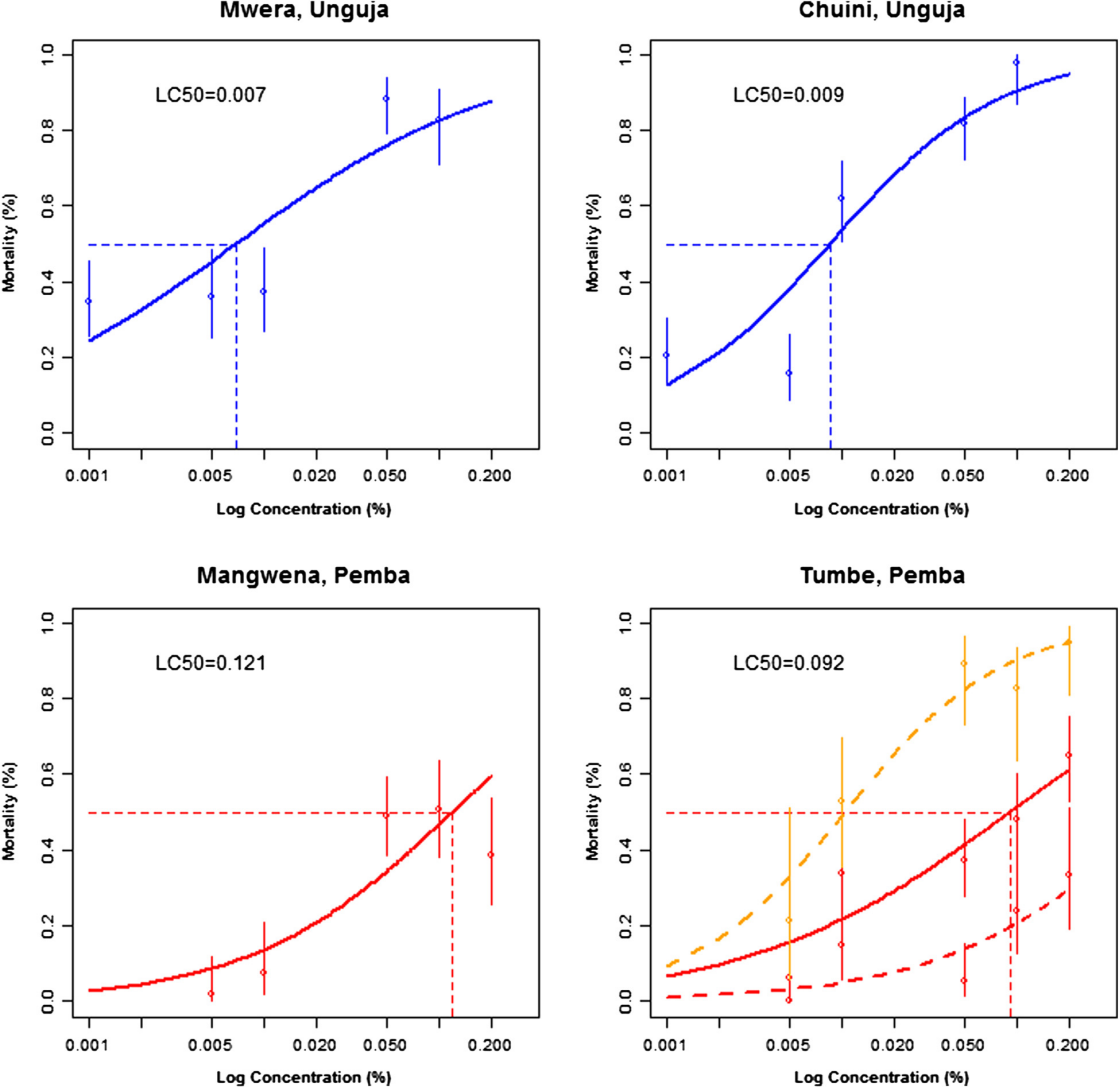
**Dose–response curves for *An. arabiensis *exposed to lambda-cyhalothrin on Zanzibar. ***An. gambiae* were exposed to a range of concentrations of lambda-cyhalothrin in WHO susceptibility tests. The lethal concentration for 50% mortality (LC_50_) was calculated by fitting a generalised linear model (GLM) using a binomial logit-link function. The LC_50_ is shown above each plot. For Tumbe, two additional curves are shown specific for *An. arabiensis* (red dashed) and *An. merus* (orange dashed).

Following the emergence of pyrethroid resistance on Pemba [17] we generated lambda-cyhalothrin LC_50_ curves from two sites on each island to quantify the scale of the resistance phenotype between the islands (Figure 2; Additional file 2). The LC_50_s from Pemba ((Mangwena = 0.121% (95% CI = 0.080-0.184) and Tumbe = 0.092% (95% CI = 0.060-0.141%)) were a magnitude greater than those from Unguja ((Mwera = 0.007% (95% CI = 0.005-0.010%) and Chuini = 0.009% (95% CI = 0.007-0.011%)) giving resistance ratios of between 10 and 17-fold. A re-assessment of the data from Tumbe showed that *An. merus* were markedly more sensitive to lambda-cyhalothrin displaying an LC_50_ closer to that of *An. arabiensis* from Unguja (LC_50_ = 0.010%; 95% CI = 0.006-0.018%) (Figure 2).

**Figure 3:**
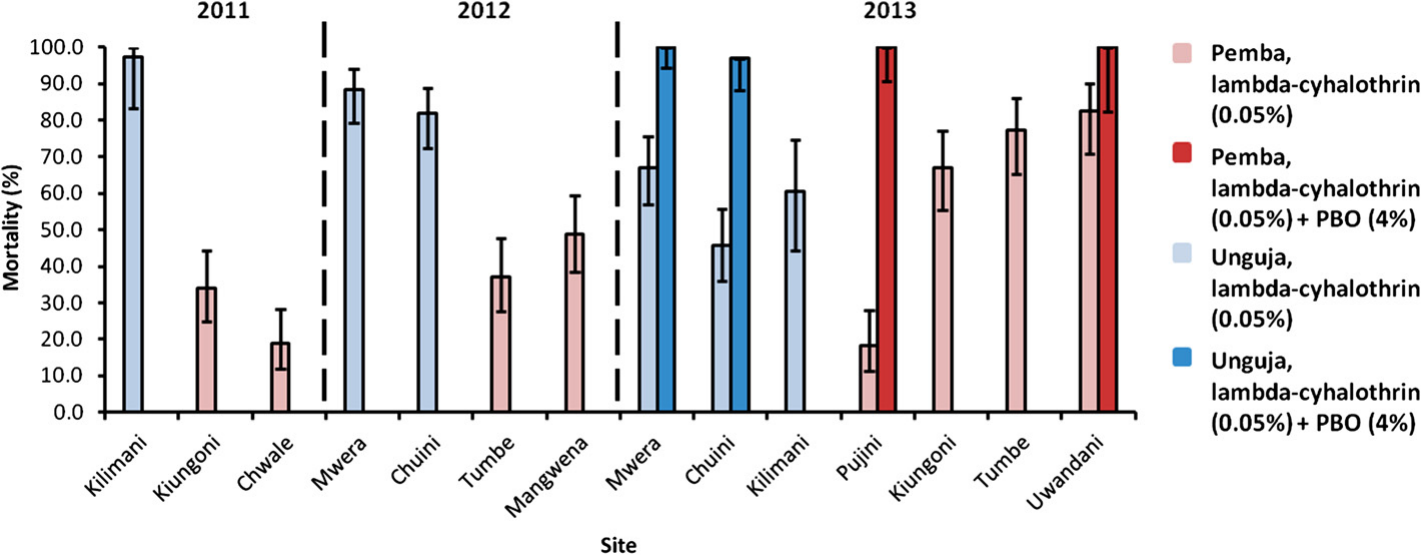
**Susceptibility of *An. gambiae *to lambda-cyhalothrin in Zanzibar between 2011 and 2013.** Mosquitoes were exposed to lambda-cyhalothrin (0.05%) in WHO susceptibility tests between 2011 and 2013. Blue (Unguja) and red (Pemba) percentage mortalities with 95% binomial confidence intervals are given one-hour of exposure to insecticide. Darker bars represent synergist assays using one-hour pre-exposure with piperonyl butoxide (PBO) performed in 2013.

In addition to the dose–response curves 1125 *An. gambiae* were tested against the WHO diagnostic dose of lambda-cyhalothrin (0.05%) in susceptibility bioassays in 2011 and 2013 (Figure 3; Additional file 2). WHO-defined resistance (less than 90% mortality [18]) was observed throughout the study in all sites from Pemba (Figure 3). The lowest mortalities observed were from Chwale in 2011 (19.0%; 95% CI = 12.1-28.3) and Pujini in 2013 (18.3%; 95% CI 11.3-27.9%). Although these data indicate that resistance remains particularly strong on Pemba, significantly higher mortality levels were observed in three out of the four sites tested on Pemba in 2013 compared with Pujini (χ^2^ p < 0.001 for each pair-wise comparison). This suggests that resistance may not be homogeneously expressed across the island. In Kiungoni, mortality to lambda-cyhalothrin approximately doubled between 2011 and 2013 indicating that in this site at least that resistance decreased over the three year study period.

As reported earlier [17], *An. arabiensis* from Unguja were susceptible to pyrethroids at the start of this study (2011). However, resistance had emerged on the island (81.9% - 88.4% mortality) in 2012. In the subsequent year, further reductions in mortality were observed in Chuini (45.7%; 95% CI 36.1-55.7%) and Mwera (67.0%; 95% CI 57.1-75.6%) confirming that resistance had arisen during the course of the study.

Synergist assays with piperonyl butoxide (PBO 4%), a general inhibitor of P450 monooxygenases, completely restored susceptibility to lambda-cyhalothrin in Unguja and Pemba (mortality = 96-100%) (Figure 3). This suggests that P450s may contribute towards the resistance phenotype we observe in *An. arabiensis*. Unfortunately, due to a restricted number of insects available, we were only able to perform one set of assays using DDT but the 2011 data do suggest potential cross-resistance in Pemba (Kiungoni, 78.8% mortality).

Finally, the carbamate bendiocarb has recently replaced lambda-cyhalothrin in IRS on Zanzibar [17] and our bioassay data show that all mosquitoes tested to date are completely susceptible to this insecticide (100% mortality, Unguja N = 57; Pemba N = 192; Additional file 2).

### Existing target-site markers for pyrethroid resistance

A sub-sample of *Anopheles* were screened for the *kdr* target site mutations (1014 F and 1014S) and *ace-1R*. None of these known resistance alleles were found over the three year period (Unguja N = 120; Pemba N = 251). Sequencing exon 20 of the sodium channel which harbours target-site mutations in other insects [30] failed to identify any additional non-synonymous mutations suggesting that alternative mechanisms underlie the pyrethroid resistance phenotype in *An. arabiensis* from Zanzibar.

### Gene expression in An. arabiensis from Pemba Island

A direct comparison of the adult transcriptome between *An. arabiensis* collected from Pemba in 2011 and a susceptible laboratory colony originating from Mozambique (MOZ) yielded 2214 probes significantly differentially expressed (1071 up (48.3%); 1143 down (51.7%)) (Additional file 3). This large number of probes and the extremely high fold changes at the ends of the expression distribution most probably reflect genetic divergence between the field and laboratory strains. We therefore do not consider this experiment in isolation; however, under the assumption that the mechanism responsible for pyrethroid resistance was the same in 2011 and 2012, this data set was combined with a second experiment conducted in 2012 on *An. arabiensis* from Pemba (PEM), Unguja (UNG) and pyrethroid-susceptible mosquitoes from Dar es Salaam (DAR). Using the 2214 probes from the 2011 experiment as our initial probe list, we subsequently filtered probes based on the hypothesis of significance in each pair-wise comparison (false discovery rate (FDR) adjusted p-value < 0.05) and greater expression in the resistant strain PEM compared to UNG and DAR (N = 2645). This left just 268 probes representing 208 unique transcripts (Additional file 3).

**Figure 4:**
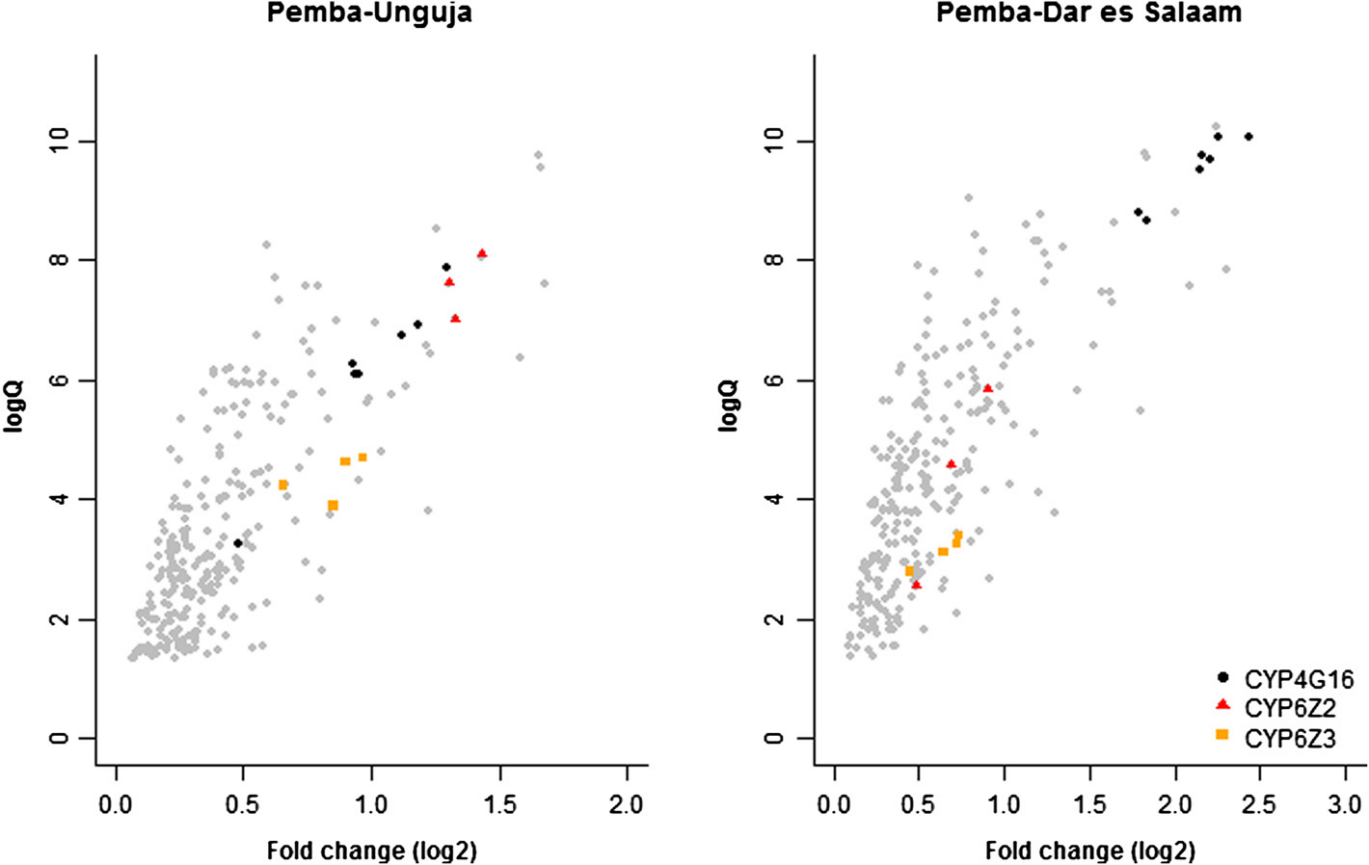
**Volcano plots for expression between *An. arabiensis *from Pemba against samples from Dar es Salaam and Unguja.** Expression plots for the 268 candidate probes significantly up-regulated in (i) *An. arabiensis* from Pemba collected in 2012 against UNG and (ii) DAR. Probes for the three candidate P450s are highlighted on each plot.

The most over-expressed transcript in the PEM vs DAR comparison was *CYP4G16* (AGAP001076-RA) (fold-change (FC) = 5.4). *CYP4G16* has four alternatively spliced transcripts and probes specific for each of these were among those most highly expressed in PEM against DAR (average FC = 4.4) and UNG (FC = 2.0) (Figure 4).

The most over-expressed transcripts in PEM compared to UNG included a Niemann-pick type c gene (AGAP002852-RA), two transcripts for enzymes involved with 5’ nucleotidase activity (AGAP005457-RA & AGAP005458-RA) and the alkaline phosphatase (AGAP001684-RA) and the P450 *CYP6Z2* (AGAP008218-RA) (Additional file 3). Two members of the *CYP6Z* family of P450s were overexpressed in all three PEM comparison and showed higher expression against UNG than samples from DAR; *CYP6Z2* (FC = 2.6 vs. 1.6) and *CYP6Z3* (FC = 1.9 vs. 1.6) (AGAP008217-RA). Members of this class of *CYP* genes have been associated with pyrethroid resistance previously [31,32].

Functional annotation of the 268 candidate probes with the DAVID analysis software tool [33] yielded no significantly enriched biological terms. Under the stringent filtering strategy used here there is a strong possibility of missing genes or biological processes involved with the resistance phenotype. Therefore, we ran a DAVID analysis on common probes over-expressed in the resistant mosquitoes from the 2012 experiment only (N = 2645) (Additional file 3). Following multiple hypothesis testing the most enriched cluster of genes was associated with P450 metabolism (enrichment score (ES) = 4.55), mitochondrial processes (ES = 3.93) and ribosomal genes (ES = 3.36) (Additional file 4). A look at individual genes solely from the 2012 analysis found that no additional P450s were contained within the PEM over expressed subset than those already described above.

### Validation of candidate gene expression in Zanzibar

Independent validation of expression data from high-throughput transcriptomic studies is a necessary step towards identifying and verifying candidate genes. Based on our final list of candidates we took six target genes forward for reverse-transcription quantitative PCR (RT-qPCR); the P450s *CYP4G16*, *CYP6Z2* and *CYP6Z3* along with three genes with putative involvement as part of a hydrocarbon synthesis pathway, acyl-coa thioesterase (AGAP003848), acyl-coa dehydrogenase (AGAP005662) and 3-hydroxyacyl-coa dehydrogenase (AGAP007784).

**Figure 5:**
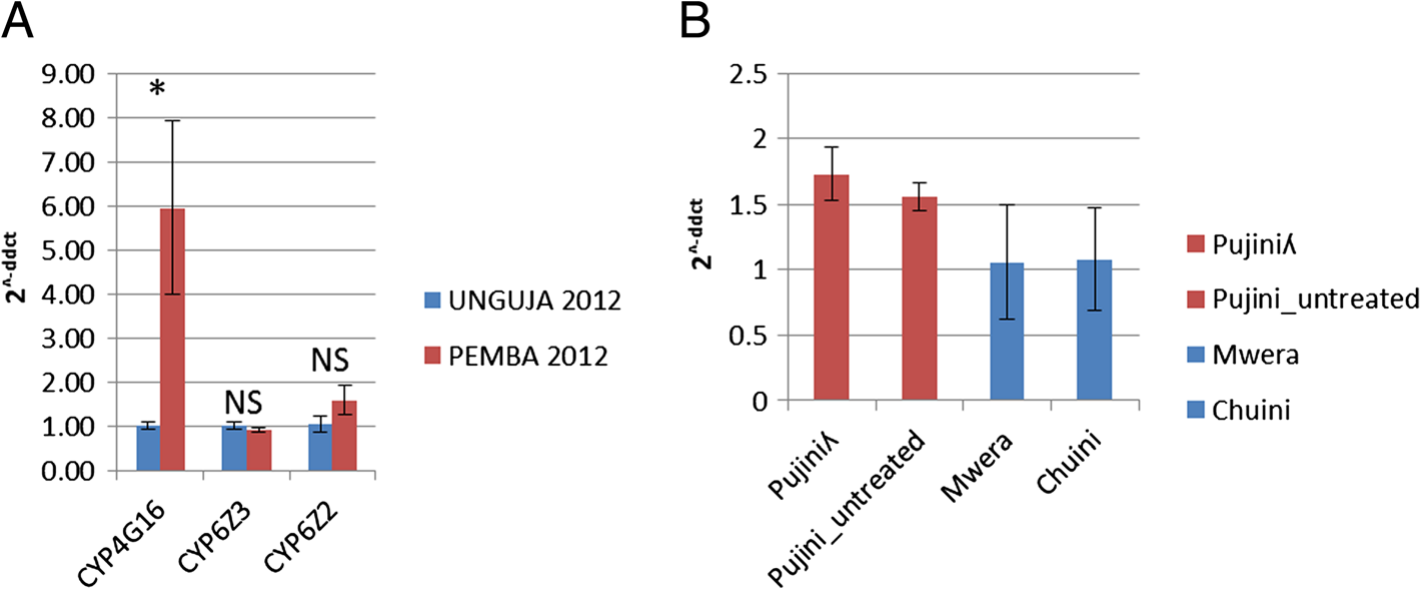
**Quantitative PCR validation of candidate P450s in *An. arabiensis *from Zanzibar. A)** Quantitative PCR (qPCR) analysis of *CYP4G16*, *CYP6Z2* and *CYP6Z3* was performed on *An. arabiensis* collected from Pemba (Mangwena) and Unguja (Chuini & Mwera) in 2012. The mean ± SEM for three ddCq values relative to Unguja are presented. **(B)***CYP4G16* expression in *An. arabiensis* collected in 2013. Four groups of mosquitoes were included in the analysis: (i) Pujini exposed to lambda-cyhalothrin (0.05%) (Pujiniλ) (ii) Pujini unexposed to insecticide (Pujini_untreated) (iii) Mwera (iv) Chuini. The mean ddCq values ± SEM of five biological replicates are presented. NS = non-significant. *p <0.05 one-sided Student’s t-test.

RT-qPCR on *An. arabiensis* collected in 2012 from Mangwena (the same site used in the microarray analysis) confirmed a significant over-expression of *CYP4G16* in Pemba compared to Unguja (ddCt = 5.5; p < 0.014) but could not validate the over-expression of *CYP6Z2* (ddCt = 1.5; p = 0.127) or *CYP6Z3* (ddCt = 0.9; p =0.206) (Figure 5A).

In 2013, we collected mosquitoes in RNAlater from two sites on Unguja (Mwera and Chuini) and from the most resistant site on Pemba, Pujini. When we compare expression against *An. arabiensis* from Mwera – the least resistant site in 2013 (mortality to lambda-cyhalothrin 67.0%) - RT-qPCR estimated a 1.6- and 1.5-fold increase in *CYP4G16* in lambda-cyhalothrin-treated and non-treated mosquitoes respectively although neither difference was significant (p > 0.05) (Figure 5B). This is approximately a quarter of the difference in expression observed between samples taken from Pemba and Unguja in 2012 (Figure 5A) and could represent the increase of pyrethroid resistance in Unguja by our sampling period in 2013.

**Figure 6:**
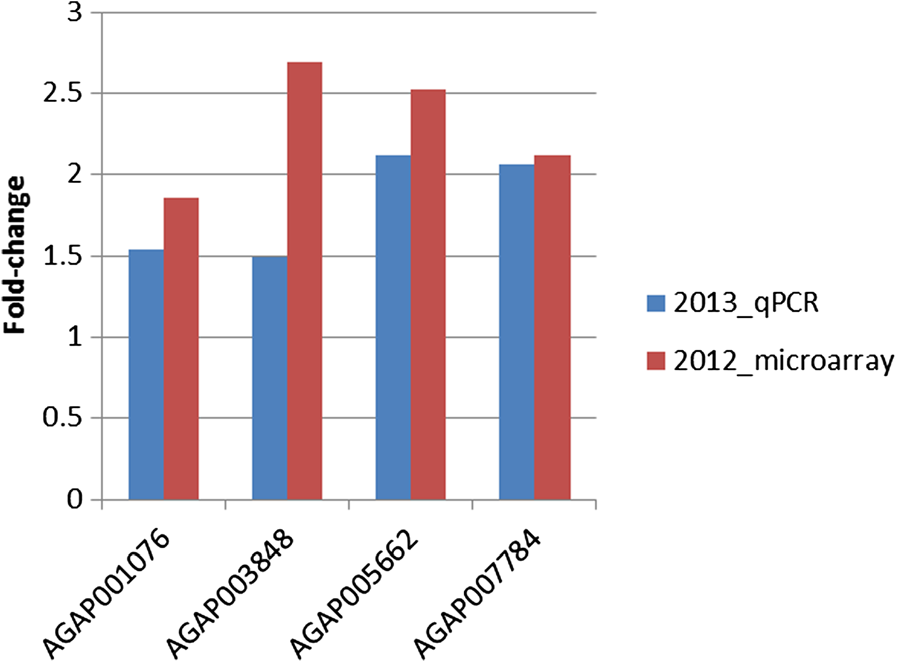
**Co-expression of *CYP4G16 *with genes significantly up-regulated in Pemba associated with fatty-acid metabolism.** The expression levels between Pemba and Unguja are compared from those in the microarrays performed on 2012 collected *An. arabiensis* samples against those from qPCR performed on 2013 samples. For the 2013 qPCR data, the fold-changes are calculated from ddCq(Pemba)/ddCq(Unguja).

Three genes with putative involvement in hydrocarbon synthesis which passed our filtering criteria in the microarray analysis (acyl-coa thioesterase, acyl-coa dehydrogenase, hydroxyacyl-coa dehydrogenase) were analysed by qPCR on *An. arabiensis* collected in 2013. The patterns of up-regulation in Pemba were extremely similar between those detected through the microarray in 2012 and those analysed by qPCR in 2013 (Figure 6). This provides further independent validation of the array experiments and suggests that these genes could be co-expressed with *CYP4G16* as part of the hydrocarbon synthesis pathway.

### Copy number variation of P450 candidates

Copy number analysis of the three major candidate genes *CYP6Z2*, *CYP6Z3* and *CYP4G16* was performed on *An. arabiensis* from Pemba surviving the higher doses of lambda-cyhalothrin compared to susceptible counterparts from Unguja. None of the candidate genes showed any significant difference between the two groups and so the increase in expression observed in the microarrays is unlikely explained by copy number changes (Additional file 5).

## Discussion

While national malaria control programmes targeting indoor resting/biting mosquitoes have enjoyed success at reducing malaria in recent years, this success has brought with it many challenges for the future of vector control including: a) the selection of physiological resistance [2], b) a change in *Anopheles* biting and resting habits [34] and c) shifts in the composition of vector species [35]. The evidence presented previously [17] and in this study suggests that at least two of these are threatening malaria control in Zanzibar.

*Anopheles arabiensis* has supplanted *An. gambiae s.s.* as the major vector in many parts of East Africa where the two species are sympatric [36,37]. In the 1960s, it was reported that *An. arabiensis* had replaced *An. gambiae s.s.* as the major vector on Zanzibar following DDT spraying as part of the Global Malaria Eradication Programme [38] although by the 1990s, *An. gambiae s.s.* had re-established itself as the main vector on Zanzibar [39]. The data presented here and in a previous publication [17] suggest that history is repeating itself, with *An. arabiensis* remerging as the dominant vector following the implementation of a scaled-up vector control programme. Pockets of *An. gambiae s.s.* and *An. merus* were found in sites proximate to the coast (e.g. Kilimani (Unguja) and Tumbe (Pemba)) but only a few *An. gambiae* (N = 7) and no *An. merus* were identified in 2013. It is worth noting that our sampling strategy was restricted to collecting larvae from breeding pools and it important to broaden these collections to collect adult vectors indoors and outdoors, and screen these for the presence of *Plasmodium* before drawing conclusions on the role of alternative vectors in contributing to the residual transmission on Zanzibar.

Pyrethroid resistance is now present throughout the entire Zanzibar archipelago. In a previous study conducted in 2010–2011, resistance was detected solely in *An. arabiensis* from Pemba suggesting that differential selective forces were acting on the two islands. However, we have subsequently shown that the proportion of mosquitoes surviving exposure to lambda-cyhalothrin (0.05%) increased approximately 3-fold in Unguja between 2012 and 2013. This rise within a single year highlights how swift the spread and selection of resistance can occur.

At present, it is difficult to determine the origins of resistance on Zanzibar. Pemba and Unguja are separated by a 50 km strip of the Indian Ocean and large numbers of human traffic moving between the two islands provide a passive route for migration of anopheline mosquitoes. Similarly, it is feasible that resistance could have emerged independently on Unguja. Resolving the genetic differentiation of *Anopheles* between Unguja and Pemba would not only provide information on the spread of resistance traits but present estimates of the diversity and effective population sizes as measures of vector control success. Regardless of the origin, resistance management strategies should be implemented equally on both islands to curb further selection.

In bioassays conducted on Pemba we observed WHO-defined resistance (less than 90% mortality) in all sites tested. Nevertheless, with the exception of Pujini it was noticeable that average resistance levels dropped between 2011 and 2013 with three out of four sites showing mortality over 67% and large increases in pyrethroid mortality in some sites (e.g. mortality in *An. arabiensis* to 0.05% lambda-cyhalothrin was 5.2% in Tumbe in 2012 but increased to 77.1% the following year). The vast majority of insects tested in these sites were *An. arabiensis* and therefore any increase cannot be attributed to species differences. It is also difficult to ascribe the differences in resistance levels across Pemba to experimental variation as we were careful to record the age of mosquito, as well as room temperature and humidity throughout – factors which can greatly influence mortality in bioassays [18].

It is possible that the change in insecticide use in IRS, from pyrethroids to carbamates may have contributed to the reduction in pyrethroid resistance. However, further rounds of bioassays, and an analysis of the strength of the resistance, via repeat determination of the LC50, is needed before it can be concluded that pyrethroid resistance has declined since the introduction of bendiocarb for IRS.

In response to the discovery of pyrethroid resistance on Zanzibar we set out to identify the underlying mechanisms in *An. arabiensis* to support future resistance management strategies. We performed two separate microarray experiments using stringent criteria to filter out probes based on significant over-expression in *An. arabiensis* from Pemba. This yielded just 208 transcripts and of these, replicate probes for three specific P450s (*CYP6Z2*, *CYP6Z3* and *CYP4G16*) previously implicated in other resistance studies in *An. gambiae* were among the most over-transcribed genes.

The P450, *CYP4G16* was up-regulated in resistant *An. arabiensis* approximately 4.5- and 2.0-fold compared to DAR and UNG respectively. Although these are fairly modest changes in expression they were among the highest observed in the comparison of the field populations. qPCR analysis gave an estimated 6-fold difference between *An. arabiensis* from Pemba compared to Unguja. Elevated expression levels of this enzyme were observed in families of *An. arabiensis* displaying an increased tolerance to deltamethrin in Northern Cameroon [40]. A modest fold-change increase was observed in a laboratory resistant strain of *An. arabiensis* from Sudan using qPCR although this was not backed-up by the microarray data in the same study [41].

*CYP4G16* is an alternatively spliced gene with four transcripts located on the X chromosome. Sequencing cloned *CYP4G16* from pooled cDNA from Pemba and Unguja revealed no SNPs between the populations and showed that *CYP4G16* is an extremely conserved enzyme between *An. arabiensis* and *An. gambiae.* The majority of insects only possess two *CYP4G* genes [42]. The other *CYP4G* gene in *An. gambiae*, *CYP4G17*, was also up-regulated in resistant *An. arabiensis* from Pemba (1.9-fold versus DAR; 1.5-fold versus UNG) but was not significantly expressed in the comparison against MOZ in 2011. *CYP4G* genes have played a key evolutionary role in the terrestrial adaptation of Insecta by catalysing the final step of cuticular hydrocarbon synthesis and thus providing a protective waterproof layer [42]. The orthologue of *CYP4G17* in *Drosophila melanogaster* (*DmCYP4G1*) catalyses an oxidative decarbonylation of long-chain aldehydes, which serves as the final step of hydrocarbon synthesis from long-chain fatty acids in insects [43]. Synthesis of cuticular hydrocarbons from long-chain fatty acids is complex and requires a suite of elongases, reductases and dehydrogenases [44]. It is therefore of interest that the final list of candidates from the microarray analysis included several genes involved in fatty-acid metabolism; acyl-coa thioesterase, acyl-coa dehydrogenase, hydroxyacyl-coa dehyrogenase, and over expression of these was confirmed in *An. arabiensis* collected in 2013 by qPCR. *DmCYP4G1* is highly expressed in the fat body and carcass of male and female *D. melanogaster* whereas the orthologue of *CYP4G16*, *DmCYP4G15*, is expressed in the thoraic-abdominal ganglion [45]*.* Tissue specific expression in *An. gambiae* shows that both *CYP4G* genes are over expressed in the insect abdomen consistent with hydrocarbon synthesis in oenocytes (Ranson *et al.* unpublished; MozAtlas [46]). In the German cockroach, *Blatella germanica*, *CYP4G19* was over-expressed five-fold in a pyrethroid resistant strain and expression was found to be greater in the abdomen than the head or thorax [47]. Cuticular-based resistance has been reported from several agricultural and medically important insects [48-50] with the over-expression of specific genes implicated in some cases [51,52]. However, whilst the up-regulation of CYP4G16 does suggest a potential role for cuticular based resistance in *An. arabiensis* in Zanzibar, it is important to recognise that the functional work to validate this hypothesis is still underway. Whether or not PBO can synergise pyrethroids (and other insecticides) by inhibiting P450s essential to cuticular formation also needs further investigation.

In contrast to target site resistance, where a single mutation can confer high levels of resistance [10] there is increasing evidence that insecticide resistance in *Anopheles* may not be underpinned by one single gene but by a suite of co-regulated enzymes in a more intricate pathway depending on the population. One example is the *CYP6Z* family in which members are over-expressed in pyrethroid resistant *An. gambiae*[31,32]. Functional work in these enzymes has found that they are unable to metabolise pyrethroids directly but instead, that they play a role in the clearance of pyrethroid metabolites. We propose that the coordinated up-regulation of multiple genes involved in CHC biosynthesis is a putative resistance mechanism underpinning the pyrethroid resistance in *An. arabiensis* in Zanzibar.

The association of P450s with resistance on Zanzibar is supported by the near full restoration of pyrethroid susceptibility we observed in the PBO assays. PBO has been incorporated into a new generation of LLINs alongside pyrethroids to combat resistance [53]. While a wider adoption of these nets as IRM tools is restricted at present, given the resistance phenotype described in *An. arabiensis* in this study (i.e. over-expression of P450s with no *kdr*), it would be interesting to determine their effectiveness against wild caught mosquitoes on Zanzibar as a possible option for resistance management.

Pyrethroid resistance in *An. arabiensis* remains patchy in both its distribution and severity in East Africa. A common phenotype in *An. arabiensis* of resistance to pyrethroids with no cross-resistance to DDT has now appeared in southern Uganda on the shores of Lake Victoria [54], in the Northern highlands of lower Moshi in mainland Tanzania [7]. There is evidence for a similar but milder resistant phenotype from Western Kenya [55]. The presence of DDT resistance on Pemba in 2011 suggests that the resistance phenotype on Zanzibar differs to that on the mainland. An additional bioassay conducted during the dry season (October) on Pemba in 2013 confirms this finding (63.0% mortality; Additional file 2) and indicates that DDT resistance has remained relatively stable between 2011 and 2013. At present there is no evidence to suggest that *kdr* plays a role in any of these regions and in this study, a large screen for 1014 F and 1014S (N = 371) as well as sequencing regions of the sodium channel where alternative *kdr* variants exist in other insect pests, provided no evidence to suggest the contrary. Whether a single mechanism of resistance in *An. arabiensis* is sweeping across the East African region or independent selection events are taking place is currently unknown. Future population genetic approaches in *An. arabiensis* from Zanzibar and a wider geographical region will no doubt provide clues on the spread of this resistance phenotype.

## Conclusion

Insecticide resistance in malaria vectors is seen as a significant hurdle to malaria elimination in Africa. There are a few well cited examples of operational failure as a consequence of resistance and experimental evidence is gathering to show that LLINs and/or IRS are not adequately killing resistant vectors [56,57]. Haji *et al.* (2013) and colleagues have already shown that resistance has a significant operational impact on the effectiveness of LLINs and IRS and warned about its potential negative effects on Zanzibar highlighting the importance of resistance management. Important proactive steps have been swiftly taken to address this issue on Zanzibar with the switch to alternative modes of action for targeted IRS. The plasticity in feeding and resting behaviour of *An. arabiensis*[58] will necessitate additional tools to control this vector. The case of Zanzibar represents an ideal opportunity to systematically monitor resistance, using entomological data and candidate molecular markers (described in this paper), and quantify its impact on our ability to control mosquito vectors.

## Competing interests

All authors declare that they have no competing interests.

## Authors’ contributions

CMJ, GD, SM and HR conceived the study. SM, KAH, BOK, JHM and CMJ planned, supervised and participated in insect collections and insecticide bioassays. JB and MD contributed to the field work and performed the molecular assays as part of their Masters' degrees, supervised by CMJ and HR. ASA facilitated the field work. BK collected and contributed insect material for microarrays. CMJ analysed the data and wrote the first draft of the manuscript. All authors read and approved the final version of the manuscript.

## Supplementary Material

Additional file 1 PCR and quantitative PCR (qPCR) primer information. Click here for file

Additional file 2 WHO susceptibility bioassay data. Click here for file

Additional file 3 Microarray gene expression data. Click here for file

Additional file 4 Cluster analysis of common candidate genes over-expressed in microarrays using DAVID software. Click here for file

Additional file 5 Copy number analysis of CYP4G16, CYP6Z2 and CYP6Z3. Click here for file
