# Supplementary material for: The dynamics of pyrethroid resistance in Anopheles arabiensis from Zanzibar and an assessment of the underlying genetic basis
Source: Parasit Vectors. 2013 Dec 6;6:343. doi: 10.1186/1756-3305-6-343 (PMC3895773; doi:10.1186/1756-3305-6-343)
Supplement: Additional file 5 — Copy number analysis of CYP4G16, CYP6Z2 and CYP6Z3. [file 1756-3305-6-343-S5.pptx]

## Slide 1
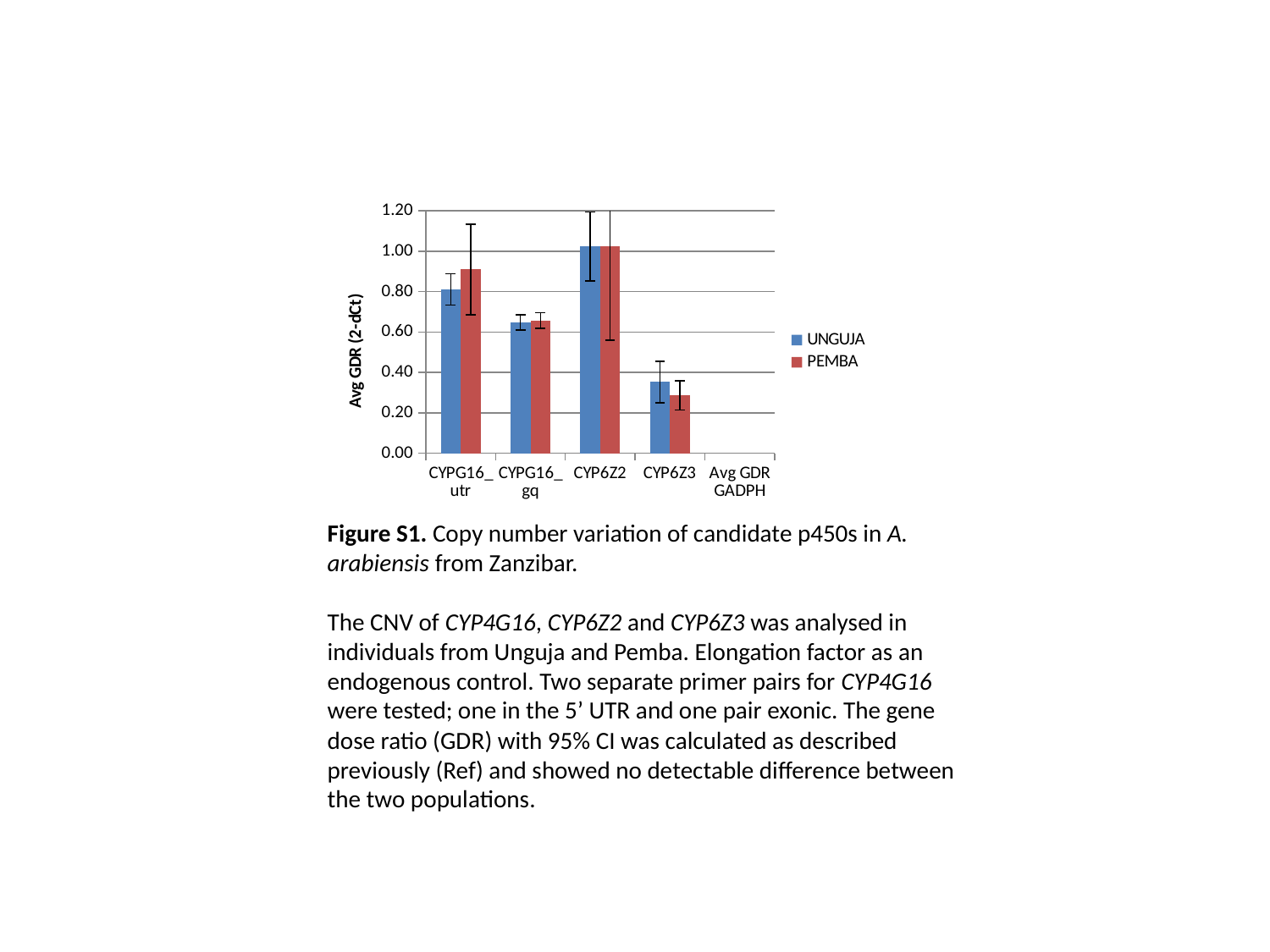

[unsupported chart]
Figure S1. Copy number variation of candidate p450s in A. arabiensis from Zanzibar.
The CNV of CYP4G16, CYP6Z2 and CYP6Z3 was analysed in individuals from Unguja and Pemba. Elongation factor as an endogenous control. Two separate primer pairs for CYP4G16 were tested; one in the 5’ UTR and one pair exonic. The gene dose ratio (GDR) with 95% CI was calculated as described previously (Ref) and showed no detectable difference between the two populations.
